# Cytoreductive surgery is feasible in patients with limited regional platinum-resistant recurrent ovarian cancer

**DOI:** 10.1186/s12957-023-03230-3

**Published:** 2023-11-30

**Authors:** Ruoyao Zou, Qidi Jiang, Xukai Luo, Mo Chen, Lei Yuan, Liangqing Yao

**Affiliations:** Department of Gynecologic Oncology, Obstetrics and Gynecology Hospital of Fudan University, Shanghai, China

**Keywords:** Cytoreductive surgery, Chemotherapy alone, Platinum-resistant ovarian cancer, Limited regional recurrent, Platinum rechallenge

## Abstract

**Introduction:**

To evaluate the efficacy of cytoreductive surgery versus chemotherapy for the treatment of limited regional, platinum-resistant ovarian cancer (PROC).

**Materials and methods:**

The clinical records of all patients with PROC treated in our center between March 2015 and March 2022 were retrospectively reviewed. We compared the oncology outcomes of patients who received cytoreduction or chemotherapy alone at relapse and presented information about postoperative adjuvant chemotherapy.

**Results:**

Among 52 patients with limited regional recurrence, 40.4% (21/52) underwent cytoreduction because of platinum resistance, and 59.6% (31/52) received chemotherapy alone. No residual disease (R0) was achieved in 20 patients (95.2%). The severe morbidity rate within 30 days after the surgery was 15%. The median follow-up was 70.6 months. Compared with the chemotherapy alone group, the surgery group with R0 had better progression-free survival (PFS) (10.6 vs. 5.1 months; hazard ratio (HR) = 0.421; *P* = 0.0035) and post-relapse survival (PRS) (32.6 vs. 16.3 months; HR = 0.478; *P* = 0.047), but there was no difference in overall survival (OS) between the two groups. Laparoscopy is associated with lesser intraoperative blood loss with no differences in survival and postoperative complications compared to the open approach (*P* = 0.0042). Subgroup survival analysis showed that compared with chemotherapy alone, surgery prolonged PFS in patients regardless of tumor size (greater than or equal to 4 cm or less). Surgery group patients who achieved R0 had an objective response rate (ORR) of 36.8% (7/19), among whom 40% (4/10) received platinum rechallenge chemotherapy and 33.3% (3/9) were administered non-platinum chemotherapy.

**Conclusion:**

When well-selected PROC patients with limited regional recurrence achieved R0, their outcomes were superior to those of patients who received only chemotherapy with an acceptable morbidity rate. Laparoscope technology could be a reliable alternative surgical approach. The reintroduction of platinum agents may be considered following surgery. Further analyses in a larger population are warranted to elucidate the risks and benefits of this surgery and adjuvant chemotherapy strategy.

**Supplementary Information:**

The online version contains supplementary material available at 10.1186/s12957-023-03230-3.

## Introduction

Epithelial ovarian cancer (EOC) is highly lethal, with approximately 295,414 newly diagnosed cases and more than 184,799 deaths annually worldwide [1]. Despite radical surgery and regular adjuvant chemotherapy, most patients with EOC experience relapse, with a median progression-free survival (PFS) of 15–18 months [2, 3]. Approximately 25% of patients experiencing first recurrence are platinum-resistant. Importantly, even in patients with initially platinum-sensitive recurrence (PSR), sensitivity to platinum-based chemotherapies decreases with each subsequent relapse and with the inevitable development of platinum-resistant disease [4]. Recently, there has been no optimal treatment regimen for these patients. Non-platinum-based monotherapy (including weekly paclitaxel, pegylated liposomal doxorubicin hydrochloride, or topotecan hydrochloride alone or in combination with bevacizumab) results in low response rates (10–15%) and short response durations (3–4 months), and the median overall survival (OS) is only approximately 12 months [5]. Therefore, determining more effective treatment strategies to improve outcomes in this uniformly fatal disease is a high priority.

Secondary cytoreduction surgery (SCS) has been attempted in PSR diseases. Three randomized phase 3 trials initiated in Germany (AGO DESKTOP III) [6, 7] the USA (GOG-0213) [8], and China (SGOG SOC-1) [9] showed that among highly selected patients, the optimal SCS group had a better PFS benefit compared to the chemotherapy alone group. Since these data support the use of SCS as a considerable and potential therapeutic option for PSR EOC, growing attention has recently been focused on the role of surgery in patients with platinum-resistant ovarian cancer (PROC). To the best of our knowledge, a few trials reported initially promising experiences in patients with isolated or low-burden relapsed disease [10, 11], whereas another retrospective study found that this strategy was employed in 96% of patients with multiple-site diffuse relapses with associated 30-day complications and mortality rates ranging up to 38% and 8%, respectively [12]. Considering the high risk of non-negligible adverse events, patients should be carefully selected for surgery, even with the observed encouraging survival data.

Regarding the selection criteria for PSR EOC, whether surgery can achieve complete resection is particularly important. The well-known DESKTOP score and the Tian model which incorporated various prognostic factors reported positive predictive values of R0 as high as 79% and 53.4%, respectively [13, 14]. However, another study had shown that patients with negative scores also have R0 rates of 61% and 70%, respectively. A subset of patients who had residual lesions after the primary surgery, or with ascites at the time of recurrence, or relapsed with a high CA125 level, or combined with extra-abdominal recurrence, may also be candidates for post-recurrence cytoreduction. Consequently, further research into reasonable selection criteria is warranted so as not to prohibit patients from undergoing potential life-extending surgery [15].

Several studies have shown that the number of recurrence sites was an important factor for the prognosis of PSR EOC who undergo surgery after recurrence. Salani, et al. showed that the number of radiographic recurrence sites was an independent risk factor for OS in recurrent ovarian cancer (ROC). The median OS was 50 months for patients with 1 or 2 recurrence sites, which was significantly higher than the median OS of 12 months for patients with 3 to 5 recurrence sites [16]. Also, Schorge et al. demonstrated in a multivariate study that a number of recurrent sites were independently associated with survival, with significantly different median OS between less than 5 and 5 or more sites of the disease group (63 vs. 22 months) [17]. In addition, The Memorial Sloan Kettering Cancer Center (MSKCC) group proposed that there were significant differences in median OS of patients with single-site recurrence, multiple-site recurrence, and carcinomatosis (60 vs. 42 vs. 28 months). The organization recommended that the disease-free interval and the number of recurrence sites should be used as selection criteria for performing SCS [18].

It has been proven that the number of recurrence sites might also be the most useful and intuitive predictor for whether a cytoreductive surgery (CRS) could result in complete resection [19]. Conte et al. showed that the number of lesions was the most relevant factor associated with a successful minimally invasive SCS [20]. Gronlund et al. investigated 38 patients and found that the number of tumor disease sites was the only variable affecting surgical outcomes [21]. Moreover, Joo-Hyuk Son et al. proposed the concept of limited regional recurrence. They suggested that limited regional recurrence was the only significant predictor of SCS without residual disease. Additionally, the R0 rate based on the criteria in this study was superior when compared with the R0 rate reported in the previous study [19]. Therefore, since the number of recurrent sites seems to be the most important predictor for R0 of surgery after recurrence and consequently influence survival, our study invokes the concept of limited regional carcinoma as a simplified selection criteria to explore the clinical value of CRS in patients suffering from platinum-resistant recurrences. Also, the postoperative adjuvant chemotherapy regimens were discussed.

## Materials and methods

### Patient selection

**Fig. 1:**
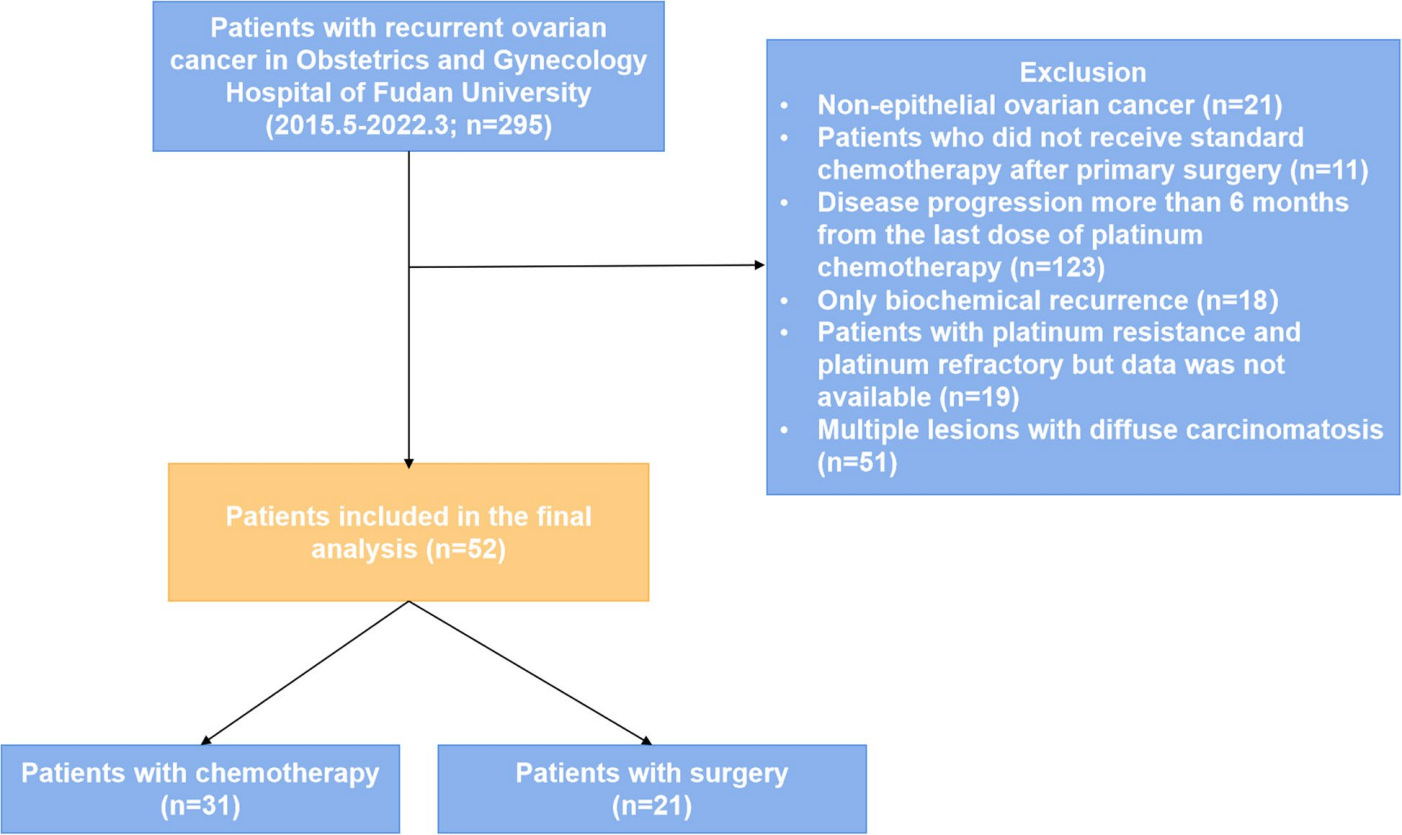
Flowchart of the patients in the study

The medical records of patients diagnosed with platinum-resistant, recurrent epithelial ovarian, fallopian tubal, or primary peritoneal cancer who were treated and followed up at the Obstetrics and Gynecology Hospital of Fudan University between March 2015 and March 2022 were retrospectively reviewed. The Ovarian Cancer Consensus Conference defined disease progression within 6 months from the last dose of platinum chemotherapy as platinum resistance and disease progression within 4 weeks from the last dose of platinum chemotherapy as platinum refractory [22]. These two types were considered in this study. The site and number of recurrences in the surgery group were calculated from preoperative imaging (PET-CT [if available], CT, or MRI) and confirmed intraoperatively. The number of recurrences in the chemotherapy group was assessed based on imaging only. We classify recurrent lesions as limited regional carcinomatosis, extra-abdominal disease, and multiple lesions with diffuse carcinomatosis. Limited regional carcinomatosis included single lesion, multiple intra-abdominal lesions (up to 3 sites) without diffuse peritoneal carcinomatosis, and limited carcinomatosis, such as localized peritoneal metastasis; other patients were defined as multiple lesions with diffuse carcinomatosis [19]. Patients meeting the following criteria were included in this study: histological diagnosis of epithelial cancer and carcinosarcoma; platinum-based chemotherapy after primary surgery; platinum-free interval (PFI) < 6 months; and radiographic evidence of recurrence, and limited regional carcinomatosis, good performance status (ECOG 0–1). Patients meeting the following criteria were excluded: non-epithelial histological condition, borderline tumors, refusal of adjuvant chemotherapy after primary surgery, only biochemical recurrence, patients with platinum-resistant and platinum-refractory but data was not available, multiple lesions with diffuse carcinomatosis (Fig. 1).

### Clinical characteristic acquisition

Patients were grouped based on surgery or chemotherapy and compared concerning clinicopathological characteristics, therapeutic regimens, and oncological outcomes. Surgical outcomes were divided into the following groups: complete resection with no residual tumor [R0], incomplete resection including residual tumor with diameter < 1 cm [R1], and residual tumor with diameter ≥ 1 cm [R2], for post-recurrence cytoreduction, R0 was considered successful cytoreduction [23, 24].

### Endpoints

The primary endpoint, PFS was defined as surgery or chemotherapy for platinum-resistant relapse to progression or death. The secondary endpoint, post-relapse survival (PRS), was defined as the interval from the diagnosis of resistant relapse to death or the date of last follow-up; OS, measured as the interval from histological diagnosis and death or the date of the last follow-up. All patients were followed up until 14 March 2023. For patients who were alive at the time of analysis, OS and PRS were censored at the time of the last follow-up. Surgical complications were assessed 30 days postoperatively according to the Clavien-Dindo classification [25]. Tumour response was evaluated according to the Response Evaluation Criteria in Solid Tumours [26].

### Statistical analyses

All statistical analyses were performed using SPSS (version 22.0) and R software (version 3.6.1). Frequency and percentage and median and range were reported for categorical and continuous variables, respectively. Baseline data among the groups were analyzed using *t*-text (continuous variables), Pearson’s chi-squared, and Fisher's exact tests (categorical variables). The Kaplan-Meier method with the log-rank test was used to estimate and compare PFS, PRS, and OS. The stratified Cox proportional hazards model was used to assess the risk factors for PFS. The estimated hazard ratios (HRs) and confidence intervals (CIs) were presented as a forest plot. The *p* value indicated statistical significance.

## Results

### Characteristics of study patients

In total, 52 out of 298 patients with relapses in our hospital were included in the final analysis, of whom 21 (40.4%) underwent surgery and 31 (59.5%) received chemotherapy alone. The R0 was achieved in 20 patients (95.2%) who underwent surgery. Initially, the analysis focuses on patients who were completely resected and those who received only chemotherapy.

The baseline demographic and clinical characteristics at the time of first-line therapy are presented in Table 1. The median age was lower in the surgery group than in the chemotherapy group, and the other features were well-balanced between the two groups. In the entire cohort, the majority of patients were diagnosed with high-grade serous (78.4%) and advanced (84.3%) ovarian cancer. Forty-one (80.4%) patients underwent primary debulking surgery (PDS) at diagnosis, whereas 10 (19.6%) patients received neoadjuvant chemotherapy followed by interval debulking surgery. The rate of incomplete resection after the primary surgery was 40.0% for the surgery group and 29.0% for the chemotherapy group.

**Table 1:** Patients’ characteristics at the time of first-line therapy

Characteristic (*N* = 51)	Surgery with R0 (*n* = 20)	Chemotherapy alone (*n* = 31)	*P* value
Age at diagnosis (year)			0.003*
Median (range)	49(27–62)	60(38–71)	
Site of origin			0.782
Ovary	19(95.0%)	27(87.0%)	
Fallopian tube	1(5.0%)	2(6.5%)	
Peritoneum	0(0)	2(6.5%)	
FIGO stage			0.914
I–II	3(15.0%)	5(16.1%)	
III–IV	17(85.0%)	26(83.9%)	
Histology			0.133
Serous	17(85.0%)	23(74.2%)	
Mucinous	2(10.0%)	1(3.2%)	
Clear cell carcinoma	0(0)	5(16.1%)	
Endometrioid	0(0)	1(3.2%)	
Mixed tumor	1(5.0%)	0(0)	
Sarcocarcinoma	0(0)	1(3.2%)	
Tumor grade			0.454
1–2 or missing	2(10.0%)	7(22.6%)	
3	18(90.0%)	24(77.4%)	
Surgical method at primary surgery			0.280
PDS	18(90.0%)	23(74.2%)	
NACT-IDS	2(10.0%)	8(25.8%)	
Residual disease at primary surgery			0.515
R0	12(60.0%)	22(71.0%)	
R1	7(35.0%)	6(19.4%)	
R2	1(5.0%)	3(9.7%)	

*FIGO* Federation of Gynecology and Obstetrics, *PDS* Primary debulking surgery, *NACT-IDS* Neoadjuvant chemotherapy followed by interval debulking surgery，* means significance

Basic characteristics and prognostic factors at the time of recurrence are shown in Table 2. Patients appeared homogeneously distributed between the two groups with regard to time from diagnosis to platinum-resistant recurrence, platinum-resistant type, number of recurrences before platinum-resistant, number of surgeries before platinum-resistant, CA125 level and ascites at platinum-resistant, pattern of recurrence, extra-abdominal recurrence. The largest lesion in the surgery group was up to 7 cm in diameter. The surgery group had 8 (40.0%) and 6 (30.0%) patients with known breast cancer (BRCA) status and homologous recombination deficiency (HRD) status, respectively, while the chemotherapy group had 12 (38.7%) and 6 (19.4%) patients with known BRCA status and HRD status, respectively. The chemotherapy group may have a higher wild-type BRCA rate (29.0% vs. 15.0%) but was not statistically different.

**Table 2:** Patients’ characteristics at the time of platinum-resistant recurrence

Characteristic (*N* = 51)	Surgery with R0 (*n* = 20)	Chemotherapy alone (*n* = 31)	*P* value
Time from diagnosis to platinum-resistant recurrence			0.441
Median (range), months	9.7(3.0–86.1)	9.7(2.6–96.2)	
Platinum-resistant type			0.286
Primary platinum-resistant disease	12(60.0%)	23(74.2%)	
Platinum-sensitive at first relapse	8(40.0%)	8(25.8%)	
Number of recurrences before platinum-resistant (*n*,%)			0.346
0	12(60.0%)	23(74.2%)	
1	5(25.0%)	4(12.9%)	
2	2(10.0%)	4(12.9%)	
3	1(5.0%)	0(0)	
Number of surgeries before platinum-resistant (including primary surgery)			0.668
1	16(80.0%)	25(80.6%)	
2	3(15.0%)	6(19.4%)	
4	1(5.0%)	0(0)	
CA125, median (range), U/ml	75.9 (3.7–1000.0)	169.3(6.8–5000.0)	0.213
Ascites at recurrence			0.486
Absent	19(95.0%)	28(90.3%)	
Present	1(5.0%)	3(9.7%)	
Pattern of recurrence			0.471
Only peritoneum^a^	14(70.0%)	21(67.7%)	
Only limphnode^b^	1(5.0%)	3(9.7%)	
Only parenchyma^c^	0(0)	3(9.7%)	
Mixed	5(25.0%)	4(12.9%)	
Mixed exclude lymph node	0(0)	2(6.5%)	
Mixed exclude parenchyma	3(15.0%)	2(6.5%)	
Extra-abdominal recurrence			0.287
Absent	17(85.0%)	30(96.8%)	
Present	3(15.0%)	1(3.2%)	
Maximum recurrent lesion size, median(range), cm	3.0(2.0-7.0)	3.0(0.9-7.0)	0.398
Platinum re-treated after platinum resistance			0.193
Yes	10(50.0%)	11(35.5%)	
No	9(45.0%)	20(64.5%)	
No-chemotherapy	1(5.0%)	0(0%)	
BRCA mutation			0.263
Yes	5(25.0%)	3(9.7%)	
No	3(15.0%)	9(29.0%)	
Missing	12(60.0%)	19(61.3%)	
HRD status			0.474
Positive	6(30.0%)	5(16.1%)	
Negative	0(0)	1(3.2%)	
Missing	14(70.0%)	25(80.6%)	
Maintenance treatment			-
First-line maintenance therapy only	1(5.0%)	2(6.5%)	
Maintenance therapy after recurrence only	6(30.0%)	8(25.8%)	
Both	1(5.0%)	2(6.5%)	
PARPi after PARPi	1(5.0%)	1(3.2%)	

*BRCA* Breast cancer, *HRD* homologous recombination deficiency, *PARPi* Poly ADP-ribose Polymerase Inhibitors

^a^Peritoneal lesions include Douglas nodule, pararectal nodule, hepatorenal recess nodule, paracolic gutter nodule, retroperitoneal mass, and so on

^b^Lymph node include para-aortic lymph nodes, pelvic lymph nodes, inguinal lymph nodes, hepatoceliac lymph nodes, and cardiodiaphragmatic angle lymph nodes

^c^Parenchyma includes liver parenchyma and spleen

Among the surgery patients, 95% (19/20) received postoperative chemotherapy and the other case was treated with etoposide (VP-16) and apatinib. All patients in the chemotherapy group received chemotherapy after platinum-resistant recurrence. With respect to chemotherapy regimens following platinum resistance, it is interesting to note that ten (50.0%) patients in the surgery group received platinum re-treated immediately after surgery, while 11 (35.5%) patients in the chemotherapy group received platinum re-treated after receiving multiline non-platinum chemotherapy. Despite not being significant, the platinum reintroduction rate was higher in the surgery group than in the chemotherapy group.

In the surgery group, 8 (40.0%) patients received maintenance therapy, including 2 (1.0%) patients who received first-line maintenance therapy, 7 (35.0%) patients who received maintenance therapy after recurrence and one (5%) patient who received Poly ADP-ribose polymerase inhibitors (PARPi) after PARPi. In the chemotherapy group, 12 (38.7%) patients received maintenance therapy, including 4 (12.9%) patients who received first-line maintenance therapy, 10 (32.3%) patients who received maintenance therapy after recurrence and one (3.2%) patient who received PARPi after PARPi. Maintenance therapy drugs included PARPi alone (olaparib, olaparib, and fluzoparib), bevacizumab alone, olaparib in combination with bevacizumab, apatinib alone, VP-16 plus apatinib. The other patient in the chemotherapy group received immunotherapy after multiline resistance and has now maintained stable disease for 14 months with carelizumab.

### Surgical procedures and safety

Among the 21 patients who underwent surgery, the incomplete resection rate was 4.8% (1/21). The patient had 500 ml of ascites during surgery, and the lesion was located on the ileum and diaphragm. Despite ileal resection and ileostomy, it was not possible to completely remove scattered miliary lesions on the diaphragm, so the operation reached R1.

Details of the procedure with complete resection are described in Table 3. We found that 7 patients (35%) received preoperative chemotherapy for platinum-resistant recurrence. The most frequent surgical procedures performed were Douglas nodule resection (5/20, 25.0%), two patients (10.0%) underwent intestinal resection and intestinal anastomosis, and one patient (5.0% ) underwent retroperitoneal mass resection. Lymphadenectomy was performed in 4 patients (20.0%), hepatoceliac lymphadenectomy was performed in 2 patients (10.0%), pelvic lymphadenectomy was performed in one patient (5.0%), and concomitant inguinal and pelvic and para-aortic lymphadenectomy was carried out in 1 patient (5.0%). As far as parenchymal relapse is concerned, splenectomy and partial hepatic resection were performed in 2 patients (10.0%), respectively. In addition, 1 patient (5.0%) underwent transvaginal resection of vaginal stump mas.

**Table 3:** Details of the surgical procedures with complete resection and postoperative 30-day complications (Clavien–Dindo classification)

**Characteristic**	**Surgery with complete resection (*N*=20)**
**Chemotherapy before surgery for platinum-resistant recurrence**	7(35.0%)
**Surgical approach**	
Laparotomy	9(45.0%)
Laparoscope	10(50.0%)
Transvaginal operation	1(5.0%)
**Procedure performed (some patients underwent more than 1 procedure)**	
**Peritonectomy**	
Douglas nodule resection	5(25.0%)
Pelvic wall nodule resection	3(15.0%)
Pararectal nodule resection	2(10.0%)
Vaginal stump mass resection	1(5.0%)
Paravesical nodule resection	1(5.0%)
Hepatorenal recess nodule resection	1(5.0%)
Paracolic gutter nodule resection	1(5.0%)
Hepatic capsule nodule resection	1(5.0%)
Periureteral nodule resection	1(5.0%)
Presacral nodule resection	1(5.0%)
Partial vaginectomy+partial vaginal wall resection	1(5.0%)
Retroperitoneal mass resection	1(5.0%)
Diaphragmatic peritonectomy	1(5.0%)
Iliac paravascular nodule resection	1(5.0%)
Pelvic peritonectomy	2(10.0%)
Intestinal surface nodule resection	2(10.0%)
Mesenteric nodule excision	2(10.0%)
Intestinal resection (Intestinal anastomosis)	2(10.0%)
**Lymphadenectomy**	
Hepatoceliac lymphadenectomy	2(10.0%)
Pelvic lymphadenectomy	1(5.0%)
Inguinal and pelvic and para-aortic lymphadenectomy	1(5.0%)
**Parenchymatectomy**	
Partial hepatic resection	1(5.0%)
Splenectomy	1(5.0%)
**Median surgical time (minutes, range)**	128(45-330)
**Intraoperative blood loss (ml, range)**	250(10-1800)
**Intraoperative transfusion**	6(30.0%)
**Postoperative transfusion**	1(5.0%)
**Median hospitalization (days, range)**	10(5-33)
**Admission to ICU**	1(5.0%)
**Postoperative complications (some patients underwent more than 1 complication, grade)**	
Infection (II)	2(10.0%)
Anaemia (II)	2(10.0%)
Ureterectasia (III)	1(5.0%)
Postoperative ileus (III)	1(5.0%)
Acute severe pneumonia (III)	1(5.0%)

*ICU* Intensive care unit

During the hospitalization, patients stayed for an average of 10 days (5–33), and four patients underwent 1 cycle of chemotherapy. Among patients who achieved R0, 65.0% (13/20) experienced a decrease in CA125 post-operatively, of whom 25% (5/20) experienced a decrease of more than 70%; 30% (6/20) patients had an elevation in CA125, of whom 25% (5/20) had an elevation of less than 20%; and the remaining case is unknown. In the patient with R1, CA125 decreased by only 3.8% after surgery.

Surgical complications with grade 3 or worse adverse events occurred in 3 (15.0%) of 20 patients in the surgery group. The G3–G4 surgical complications included ureterectasia, postoperative ileus, and acute severe pneumonia, which was the only case transferred to the intensive care unit. None of the patients died during the postoperative period.

In the surgery group, the laparoscopic rate was similar to the laparotomy rate, with only one patient in the laparoscopy group having ascites (approximately 10 ml) found during surgery. There was no difference between the two groups in maximum recurrent lesion size, pattern of recurrence, median surgical time, median length of hospitalization, and postoperative complication rate, except that the intraoperative blood loss was significantly lesser in the laparoscopy group than in the laparotomy group (Supplementary Table S1).

#### Survival results

**Fig. 2:**
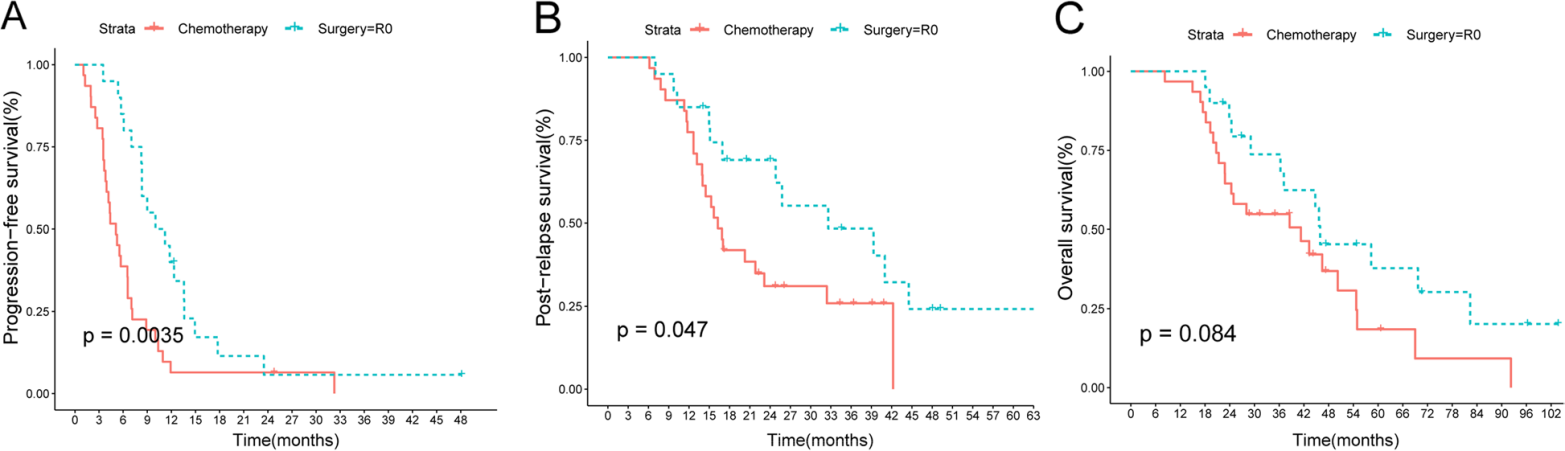
**A** PFS, **B** PRS, and **C** OS in patients receiving surgery with R0 versus chemotherapy alone

Median follow-up was 70.6 months, at which point 48 patients had disease progression (18 in the surgery group with R0 and 30 in the chemotherapy group), and 36 patients had died (13 in the surgery group with R0 and 23 in the chemotherapy group). Our survival analysis showed that patients who received R0 were associated with longer median PFS (10.6 vs. 5.1 months; HR = 0.421; *P* = 0.0035) and PRS (32.6 vs. 16.3 months; HR = 0.478; *P* = 0.047) compared to those in the chemotherapy group (Fig. 2A, B). 1-year PFS rates were 34.3% and 6.5%, respectively. The 3-year PRS rates for patients with complete resection and chemotherapy alone were 40.3% and 25.9%, respectively. However, the median OS was not significant in patients who underwent complete resection compared to the chemotherapy group (45.3 vs. 41.3 months; HR = 0.545; *P* = 0.084; Fig. 2C). Further, PFS, PRS, and OS for patients with R1 were 3.7, 3.7, and 6.3 months, respectively, all lower than the median for the chemotherapy group. When comparing the survival of different surgical approaches in the surgery group, we found no difference in PFS, PRS, and OS between the laparoscopy and laparotomy groups (Supplementary Figure S1).

**Fig. 3:**
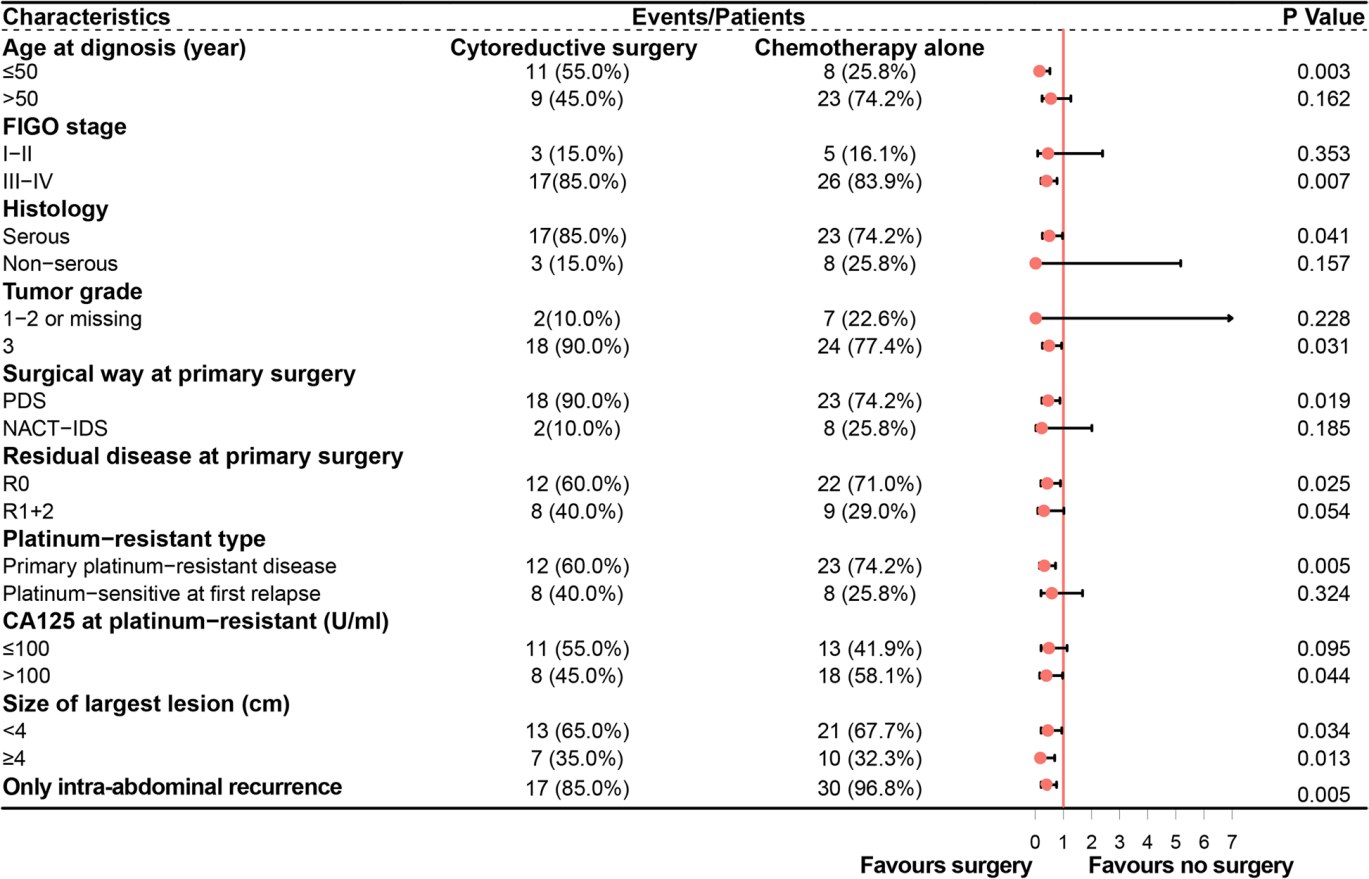
Subgroup analysis for progression-free survival in patients receiving surgery with R0 versus chemotherapy alone. Federation of Gynecology and Obstetrics, FIGO; primary debulking surgery, PDS; neoadjuvant chemotherapy followed by interval debulking surgery, NACT-IDS

In subgroups stratified by age ≤ 50 years old, FIGO stage III–IV, serous tumor, high-grade tumor, PDS at primary surgery, no residual disease (R0) at primary surgery, primary platinum-resistant disease, CA125 level > 100 U/ml at platinum-resistant recurrence, and only intra-abdominal recurrence, patients who underwent surgery with R0 showed superiority in PFS compared with the chemotherapy group (Fig. 3). It is noteworthy that PFS was beneficial in the surgery group regardless of the tumor size (greater or equal to 4 cm or smaller than 4 cm).

As for chemotherapy after surgery, we observed an objective response rate (ORR) of 36.8% (7/19) in the surgical group with R0, including 40% (4/10) after immediate platinum re-treatment, and 33.3% (3/9) after second-line chemotherapy. Table 4 lists the platinum-retreated information. In addition, there was no significant difference in PFS between the two groups (11.2 vs. 9.0 months; *P* > 0.05).

**Table 4:** Platinum-retreated status in the surgery group

Platinum-based regimen	Surgery group (*N* = 10)
Gemcitabine+oxaliplatin	1
Gemcitabine+nedaplatin	1
Paclitaxel+carboplatin	4
Paclitaxel+cisplatin	2
Paclitaxel+lobaplatin	2

## Discussion

This study reports on an exploratory attempt to perform surgery for patients with PROC at a single institution in China. We evaluated the outcomes of different treatment modalities received in patients with limited regional recurrence and explored the possibility of surgical intervention.

Several studies have demonstrated that patients with PROC receiving complete debulking have a significantly longer OS and PFS than those with residual diseases following surgery [12]. Our data indicate that one patient who failed to achieve complete resection had a significantly shorter median PFS and PRS than the surgery group with R0, and even shorter than the chemotherapy group, which is in accordance with prior studies. Accordingly, surgical indications should be strictly controlled in PROC patients. As we observed the R0 rate of more than 90% in our cohort, and complications were within an acceptable range, the limited regional recurrence could serve as an alternative criterion for selecting candidates for exploratory trials in patients with PROC.

In particular, certain patients in our surgery group underwent several cycles of chemotherapy prior to post-recurrence cytoreduction, like neoadjuvant chemotherapy before interval debulking surgery, which served to reduce or control the lesions to meet the surgical threshold. Tuninettid et al reported a similar situation [12]. The results of our study indicated that PRS was longer in the surgery group with R0 than in the chemotherapy group. According to these findings, even ineligible patients with excessive tumor burden may receive several cycles of chemotherapy before being evaluated, at which point surgery could be performed if complete resection is possible.

Similar to the rationale for initial primary cytoreductive surgery, the benefits of surgery for patients with PROC may be that by removing tumor lesions that have developed resistance, the residual tumor cells have a higher growth rate and tend to be better perfused with chemotherapeutic agents, which may make them more sensitive to chemotherapy [27–29]. Our results support this conjecture, with patients who received chemotherapy after surgery with R0 having a significantly longer PFS than the chemotherapy alone group. Moreover, this advantage was also observed in patients with lesions larger than 4 cm in diameter, which was possibly caused by a relatively weak density of chemotherapeutic drug perfusion in larger lesions. Consequently, if a localized tumor with a large tumor volume is resectable, surgery could be considered. Even though the OS analysis of our data did not show a significant difference, there was a trend towards a better outcome in the surgery group as compared to the chemotherapy group, and a larger sample size may be necessary to provide a more compelling conclusion.

Previous research has demonstrated that the anatomic site of relapse appears to have a substantial effect on survival. In a prior study, patients with localized lymph node recurrence who underwent SCS with R0 had a median PRS of 63 months, which was substantially longer than patients with localized peritoneal recurrence (41 months) and patients with localized parenchymal recurrence (24 months) [30]. Among patients with PSR EOC who underwent salvage lymphadenectomy, median PFS in patients with isolated lymph node recurrence was superior to the patients with lymph node recurrence together with other sites of disease (27 months vs. 12 months) [31]. In addition, biological characteristics of ovarian tumors, such as the status of BRCA mutations, have been reported as potential selection criteria for SCS, however, their role remains unclear. For the oncological outcomes after hepatic resection (HR) in PSR EOC, the 3-year PFS rate of BRCA-mutated patients was 81.0%, which was considerably higher than that of wild-type patients (15.0%), suggesting that BRCA mutation status could facilitate treatment decision making for ROC patients who received HR [32]. In contrast, the BRCA mutational status did not affect the clinical outcome of salvage lymphadenectomy as SCS [31]. The recurrence pattern and genetic status of our entire cohort were comparable between the surgery and chemotherapy groups. No selection bias was present in our results. Due to the fact that 70% of our patients received their initial diagnosis prior to 2018 when genetic testing was uncommon, we were unable to analyze the impact of genetic mutations on the outcome of surgery following platinum resistance using the available data. Future studies are required to focus on the molecular hereditary characteristics of OC. We anticipate additional evidence supporting the benefits of surgery after recurrence, taking BRCA status into account, in order to personalize treatment strategies for specific categories of populations.

Several studies have demonstrated that minimally invasive surgery (MIS) is a feasible and safe method for achieving optimal SCS in certain platinum-sensitive patients. This approach has favorable perioperative outcomes compared to the open approach without compromising survival [20, 33, 34]. At the same time, robotic-assisted surgery can also be used as an approach for specific ROC patients in the absence of carcinomatosis [35]. In our situation, patients with limited regional recurrence could also gain some survival benefit from minimally invasive CRS with less intraoperative blood loss.

The function of HIPEC in PROC is currently unknown. Few studies examined the efficacy of HIPEC in conjunction with CRS in patients with PROC. Bakrin N et al. prospectively performed CRS together with HIPEC for 62 chemoresistant ROC patients, suggesting no significance in the median OS of the chemosensitive and chemoresistant group (52 months vs. 48 months) [36]. The same study group confirmed this finding in the subsequent multi-centered retrospective analysis, which included 223 platinum-resistant patients. Their results showed in those who received R0 surgery and HIPEC, there was no significant difference in the median OS of the chemosensitive and chemoresistant group (51.6 months vs. 47.2 months) [37]. Besides, the randomized trial of Spiliotis J et al. suggested significantly longer OS in the HIPEC+CRS group than the non-HIPEC+CRS group. In their subgroup analysis of the HIPEC+CRS group, the OS of the platinum-resistant and platinum-sensitive group was comparable (26.8 vs. 26.6 months), while in the non-HIPEC+CRS group, the OS of the platinum-sensitive was significantly longer than platinum-resistant group (15.2 vs. 10.2 months) [38]. All of these studies indicated that the addition of HIPEC to CRS is crucial for minimizing the survival gap between platinum-resistant and platinum-sensitive patients. However, Ayhan, A. et al. stated that in a subgroup analysis of ROC patients undergoing CRS plus HIPEC, the median PFS (21 months) was significantly higher in platinum-sensitive patients than in platinum-resistant patients with a median PFS of 6 months. Platinum resistance was found to be a negative prognostic factor for PFS [39]. Similarly, the study of Jian-Hua Sun et al. suggested for patients receiving CRS plus HIPEC, the median OS of platinum-sensitive patients was significantly longer than platinum-resistant patients (65.3 vs. 20.0 months) [40]. No patient in our case experienced postoperative HIPEC. Due to the undefined benefit of CRS+HIPEC for patients with PROC, we must contemplate this treatment with caution. Future large-scale research is required to elucidate the clinical utility of CRS+HIPEC in patients with PROC.

We observed that platinum re-treated was attempted in both the surgery and chemotherapy groups. Previously, continuous single-agent non-platinum chemotherapy was considered a standard of care in PROC patients. Considering platinum-based therapy's toxicity and the palliative treatment goal for patients with PROC, a non-platinum-based, low-toxicity approach may be more appropriate for patients in poor physical condition and who have poor responses to platinum-based therapy. Despite this, platinum has consistently been identified as the most effective chemical agent for treating EOC as evidenced by numerous studies exploring the benefits of platinum re-treatment. At the 2022 ASCO meeting, a meta-analysis of 157 studies that included 6327 patients indicated that patients with PROC can benefit significantly from the reintroduction of platinum-based chemotherapy with a response rate (RR) of 36% for platinum-based chemotherapy compared with 16% for non-platinum-based chemotherapy [41]. According to this research, platinum-containing regimens are included in the 2023 NCCN guidelines as “other recommended regimens" and "potentially effective regimens” for platinum-resistant relapses, but not for platinum-refractory disease [42]. When it comes to the mechanism of platinum re-treatment, some scholars have reported that patients with PROC can still benefit from platinum treatment after an interval of non-platinum treatments has been administered and that patients treated with carboplatin had significantly improved OS. Possibly, this occurs because prolonged platinum-free intervals allow for the loss of platinum resistance in the tumor [43, 44]. Alternatively, new data on epigenetic changes during tumor progression and the use of epigenetic therapy suggest that epigenetic modifications that contribute to chemotherapy resistance have the potential to be reversed by epigenetic therapy [45]. Hypomethylating agents such as azacitidine, decitabine, or gemcitabine can induce the re-expression of epigenetically silenced genes and reverse the carboplatin resistance of EOC cells. The combination of one of these agents and platinum showed a promising ORR (22–37%) and clinical benefits in patients with PROC [46–48]. These data provide more evidence in favor of reintroducing platinum chemotherapy. Based on our study, platinum treatment was re-administered more frequently after surgery group as compared with only the chemotherapy group (50.0% vs. 35.5%). Surgeons may be more willing to try platinum-based drugs because surgery reduces the burden on platinum-resistant lesions, which may enhance the response to platinum-based therapy. The ORR following platinum-based and non-platinum-based treatment after surgery was both greater than 35%, which is higher than previously reported ORR for only non-platinum regimens, with the platinum-based treatment group performing better than the non-platinum group (40% vs. 33.3%). However, there was no difference in PFS between the two groups. Despite more evidence being needed to explore these answers, our study on platinum rechallenge is consistent with the results of the appeal meta-analysis and offers treatment options for surgical patients with PROC who can tolerate platinum therapy.

The statistical results of the comparison between the two treatment modalities support the potential benefits of surgery in patients with localized recurrence. To the best of our knowledge, this is the first study to investigate the possibility of platinum reintroduction after surgery in patients with PROC. This study has some limitations. First, in retrospective design, there still exists an inherent bias in the interpretation of preexisting data and the selection of surgical patients. As the lesions in the chemotherapy group were only assessed by imaging and not verified by surgical pathology, these patients may have had more extensive lesions and their tumor loads may have been greater than those in the surgery group. Second, the small sample size of a single institution limits the generalization of the conclusions. Equally important, this study was unable to incorporate the potential effect of salvage chemotherapy regimens and maintenance therapy on survival due to its diversity. Finally, despite the low postoperative complication rates, we did not describe the beneficial or detrimental effects of the two therapeutic strategies and the different postoperative adjuvant chemotherapy regimens on quality of life to provide more convincing evidence of safety.

## Conclusions

Our findings demonstrate that the therapeutic strategy aimed at achieving complete resection appears feasible in well-selected patients with limited regional recurrence with PROC. Laparoscopy may also be an option. Providing appropriate preoperative chemotherapy may also increase the number of patients who are eligible for inclusion. Additionally, platinum-based chemotherapy may be considered as part of the postoperative chemotherapy strategy. For platinum-resistant diseases, future prospective randomized trials involving larger samples from multiple institutions are necessary to eliminate subjective factors, increase the predictability of complete cytoreduction, and provide patients with strategic options with potential benefits.

### Supplementary Information

**Additional file 1: Supplementary Figure S1.** (A) PFS, (B) PRS, and (C) OS in patients undergoing laparoscopy versus laparotomy in the surgery group.

**Additional file 2: Supplementary Table S1.** Surgical details and postoperative 30-day complications (Clavien–Dindo classification) based on the surgical approach.

## Data Availability

The datasets used and/or analyzed during the current study are available from the corresponding author on reasonable request.
