# Supplementary figures and images for: Cytoreductive surgery is feasible in patients with limited regional platinum-resistant recurrent ovarian cancer
Source: World J Surg Oncol. 2023 Nov 30;21:375. doi: 10.1186/s12957-023-03230-3 (PMC10688147; doi:10.1186/s12957-023-03230-3)

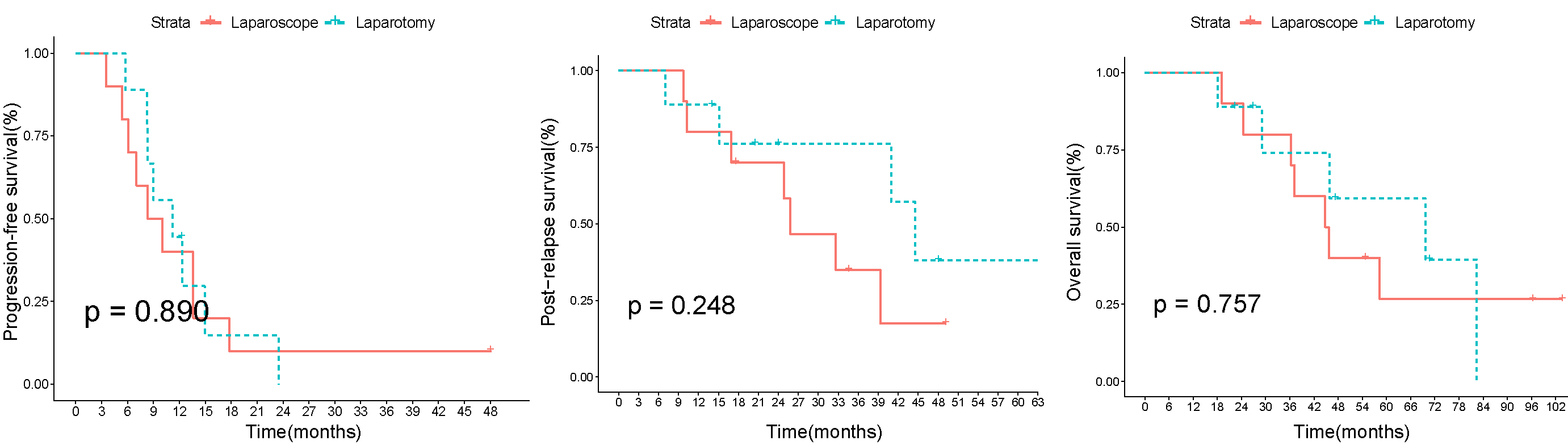

Supplement: Supplementary file 1 — Additional file 1: Supplementary Figure S1. (A) PFS, (B) PRS, and (C) OS in patients undergoing laparoscopy versus laparotomy in the surgery group. [file 12957_2023_3230_MOESM1_ESM.tif]
