# Supplementary material for: Cytoreductive surgery is feasible in patients with limited regional platinum-resistant recurrent ovarian cancer
Source: World J Surg Oncol. 2023 Nov 30;21:375. doi: 10.1186/s12957-023-03230-3 (PMC10688147; doi:10.1186/s12957-023-03230-3)
Supplement: Supplementary file 2 — Additional file 2: Supplementary Table S1. Surgical details and postoperative 30-day complications (Clavien–Dindo classification) based on the surgical approach. [file 12957_2023_3230_MOESM2_ESM.docx]

**Supplementary Table 1**

Surgical details and postoperative 30-day complications (Clavien–Dindo classification) based on the surgical approach.

| **Characteristic** | **[Laparoscope](javascript:;)**  **(n=10)** | **[Laparotomy](javascript:;)**  **(n=9)** | **P value** |
| --- | --- | --- | --- |
| **Maximum recurrent lesion size**  Median(range), cm | 3(2-6) | 3.5(2-7) | 0.455 |
| **Pattern of recurrence**  Only Peritoneum  Only Limphnode  Only Parenchyma  Mixed | 7 (70.0%)  1(10.0%)  0(0)  2(20.0%) | 6(66.7%)  0(0)  0(0)  3(33.3%) | 1.0 |
| **Median surgical time** (minutes, range) | 116(45-310) | 170(72-330) | 0.265 |
| **Median hospitalization** (days, range) | 9(5-33) | 10(7-15) | 0.950 |
| **Intraoperative blood loss** (ml) | 100(20-225) | 600(400-850) | **0.0042*** |
| **Postoperative complications**  All grades  Grade≥3 | 2(20.0%)  2(20.0%) | 4(44.4%)  1(11.1%) | 0.350  1.0 |
